# Scale-Up of Capsular Polysaccharide Production Process by *Haemophilus influenzae* Type b Using k_L_a Criterion

**DOI:** 10.3390/bioengineering9090415

**Published:** 2022-08-25

**Authors:** Omar Pillaca-Pullo, Lucas Dias Vieira, Mickie Takagi

**Affiliations:** 1 Centro de Investigación en Biodiversidad para la Salud, Universidad Privada Norbert Wiener, Lima 15046, Peru; 2 Laboratório de Desenvolvimento de Processos, Divisão de Desenvolvimento e Inovação, Instituto Butantan, Av. Vital Brasil, 1500, São Paulo 05503-900, Brazil

**Keywords:** *Haemophilus influenzae*, polysaccharide, k_L_a, batch mode, fed-batch mode

## Abstract

Polyribosyl-ribitol-phosphate (PRP) from *Haemophilus influenzae* type b (Hib) is an active immunizing molecule used in the production of the vaccine against *H. influenzae*, and industrial production could contribute to satisfying a world demand especially in developing countries. In this sense, the aim of this study was to establish a scale-up process using the constant oxygen mass transfer coefficient (k_L_a) such as the criterion for production of PRP in three different sizes of bioreactor systems. Three different k_L_a values (24, 52 and 80 h^−1^) were evaluated in which the biological influence in a 1.5 L bioreactor and 52 h^−1^ was selected to scale-up the production process until a 75 L pilot-scale bioreactor was achieved. Finally, the fed-batch phase was started under a dissolved oxygen concentration (pO_2_) at 30% of the saturation in the 75 L bioreactor to avoid oxygen limitation; the performance of production presented high efficiency (9.0 g/L DCW-dry cell weight and 1.4 g/L PRP) in comparison with previous scale-up studies. The yields, productivity and kinetic behavior were similar in the three-size bioreactor systems in the batch mode indicating that k_L_a is possible to use for PRP production at large scales. This process operated under two stages and successfully produced DCW and PRP in the pilot scale and could be beneficial for future bioprocess operations that may lead to higher production and less operative cost.

## 1. Introduction

*Haemophilus influenzae* type b (Hib) is a fastidious Gram-negative capsulated or non-capsulated bacterium, coccobacillus, that presents a filamentous or pleomorphic shape [1]. The invasive diseases in childhood cause meningitis, pneumonia and other rarer forms of diseases, such as epiglottitis, septicemia, septic arthritis, bacteremia and cellulitis [2]. There are six capsular serotypes (a–f) where type b is responsible for more than 95% of systemic infections [3]. This exopolysaccharide located in the outer region of the cell is the main factor of virulence and is composed by repeated units of the polyribosyl-ribitol-phosphate (PRP) through phosphate diester linkages [4,5]. The exopolysaccharide PRP is an antigen that is attractive to formulate vaccines against Hib after reaching high purity in the downstream and subsequent conjugation [6,7,8,9]. Exopolysaccharide PRP is the T-cell independent antigen; therefore, it cannot be presented to T cells, while B cells can recognize polysaccharides through superficial IgM. When B cells bind to a conjugated polysaccharide, i.e., a carrier protein is attached chemically to the polysaccharide to stimulate a stronger and longer immunity response, this protein provides epitopes of T cells and triggers some reaction that helps B cells to proliferate and start the affinity maturation process, and establish memory [3,10,11,12]. In this sense, the vaccine allowed the control of Hib infections worldwide despite that population in developing countries, especially children, not having access to the vaccine in time [1,13].

Vaccines and several products with therapeutical applications are part of the global bioprocess market to be valued at USD 360 million by 2024 [14]. Due to great demand, this bioprocess should reach industrial scale under three development stages (laboratory or bench scale, pilot scale and full-scale manufacturing) [15,16]. In the case of PRP production for vaccine manufacturing, it requires a bioprocess developed to achieve a desirable productivity into a bioreactor of high operation volume. However, the bioprocesses are difficult to standardize and commonly limited by the scaling process because they did not reach the required efficacy [14]. Moreover, the large-scale process faces technical challenges correlated with the cell source, culture media, use of pure reagents as precursors, sterilization step, type of product and unit operations [14,17,18]. In this sense, *H. influenzae* requires a complex culture medium containing Hemin and NAD used as growth factors for aerobic conditions, which impact the process costs and product recovery [12,19]. For this reason, improving processes of PRP production (up-stream) as well as effective scale-up and purification (down-stream) could contribute to achieving a cheaper and more competitive formulation of conjugate vaccines in a large-scale process [20].

There are several scale-up strategies; one efficient strategy is applying the scale-up criterion as the volumetric power input (P/VL), mixing time, impeller tip speed or constant volumetric mass transfer coefficient (k_L_a) in different size fermenters [21,22,23]. In the case of aerobic fermentation, maintaining constant oxygen transfer or k_L_a is a good choice [16]. k_L_a is a standard parameter for the characterization of mass transport by correlating the mass transfer rate with the concentration change [24]; this is the most important parameter for the design and operation of mixing/sparging [25]. As supplying adequate oxygen (gas-liquid mass transport) is a significant factor in aerobic cultures, maintaining a similar k_L_a has been frequently employed as the basis in the scaling-up process; besides that, the scale-up basis with the k_L_a criterion is commonly used in around of 30% of the fermentation industry [23,26,27]. The aim of this work was to demonstrate an efficient use of the k_L_a criterion in the scale-up process through three different sizes of bioreactor systems for the production of PRP by Hib.

## 2. Materials and Methods

### 2.1. Microorganism

The *Haemophilus influenzae* type b (Hib) GB3291 strain was acquired from the Núcleo de Coleção de Microorganismos of the Instituto Adolph Lutz, São Paulo. The working seed was prepared according to Takagi et al. [19] and stored in liquid nitrogen.

### 2.2. Medium Composition

The inoculum and batch media were prepared to 1 L with: 5.0 g NaCl (Sigma-Aldrich, Saint Louis, MO, USA), 2.5 g K_2_HPO_4_ (Sigma-Aldrich, Saint Louis, MO, USA), 13.1 g Na_2_HPO_4_ (Sigma-Aldrich, Saint Louis, MO, USA), 10.0 g soytone (BD Biosciences, San Jose, CA, USA), 5.0 g yeast extract (BD Biosciences, San Jose, CA, USA), 5.0 g glucose (Merck, Darmstadt, Germany), 15.0 mg NAD (Sigma-Aldrich, Saint Louis, MO, USA) and 30.0 mg hemin (Sigma-Aldrich, Saint Louis, MO, USA). For the Fed-batch, 1 L of feed medium was prepared with 200 g yeast extract (BD Biosciences, San Jose, CA, USA) and 200 g glucose (Merck, Darmstadt, Germany). The final pH was adjusted to 7.0 with 5 M NaOH (Merck, Darmstadt, Germany) and was sterilized by filtration in a Millipore system with a pre-filter and a 0.22 μm sterile membrane (Merck Millipore, Darmstadt, Germany).

### 2.3. Inoculum

A bacterial suspension of 100 μL (1.0 × 10^10^ CFU/mL), stored at −70 °C, was transferred to an Erlenmeyer flask of 300 mL containing 50 mL of the sterile medium culture and incubated for 6 h at 37 °C without agitation under a 5–6% CO_2_ atmosphere. Approximately 25 mL (OD_540nm_ = ~0.6) of the bacterial suspension were transferred to the Erlenmeyer flask of 1 L containing 200 mL of the medium and incubated at 37 °C with agitation at 250 rpm for 14 h.

### 2.4. Evaluation in Bioreactor Systems

Two groups of experiments were carried out: (1) to evaluate and select the best k_L_a (as an initial step, several experiments were carried out in a 1.5 L bench-scale bioreactor system at different stirring speeds in order to analyze the effect of the pO_2_ level on the production capability of cells); (2) experiments in the 15 L bench-scale and 75 L pilot-scale bioreactor systems keeping the same selected k_L_a chosen for the first group under ht ebatch operation.

#### 2.4.1. Specifications of Bioreactor Systems

Cultures for PRP production were carried out in a 1.5 L bench-scale bioreactor system (Infors HT, Bottmingen, Switzerland), and the scale-up fermentations were run in 15 L lab-scale and 75 L pilot-scale bioreactor systems (Bioengineering AG, Wald, Switzerland). The specification of each bioreactor system is presented in Table 1.

#### 2.4.2. Determination of Volumetric Oxygen Mass Transfer Coefficient (k_L_a)

To determine the k_L_a values in bioreactor systems, an unsteady state gassing-out method was employed [28]. This empirical value must be experimentally assayed (typically in an abiotic setting) [29]. The liquid medium was purged with inert nitrogen gas for displacing dissolved oxygen in the medium; then, the aeration rate supply and stirring speed conditions were turned on, while the time-course saturation of the dissolved oxygen concentration (pO_2_) was monitored. The liquid balance of pO_2_ during this short time period can be expressed in Equation (1):

(1) $$\frac{dC_{L}}{dt}=\;k_{L}a\left(C_{L}^{*}-C_{L}\right)\;$$

where *k_L_* is the liquid film oxygen transfer coefficient (cm/h); *a* is the gas-liquid interfacial area per unit volume of liquid (cm^2^/cm^3^); and $C_{L}^{*}$ and *C_L_* are the saturation and local dissolved oxygen concentrations in the liquid medium (mmol/L), respectively. Consequently, the slope of this equation can be regarded as k_L_a.

#### 2.4.3. Batch Cultures in 1.5 L Bench-Scale Bioreactor System

**Figure 1:**
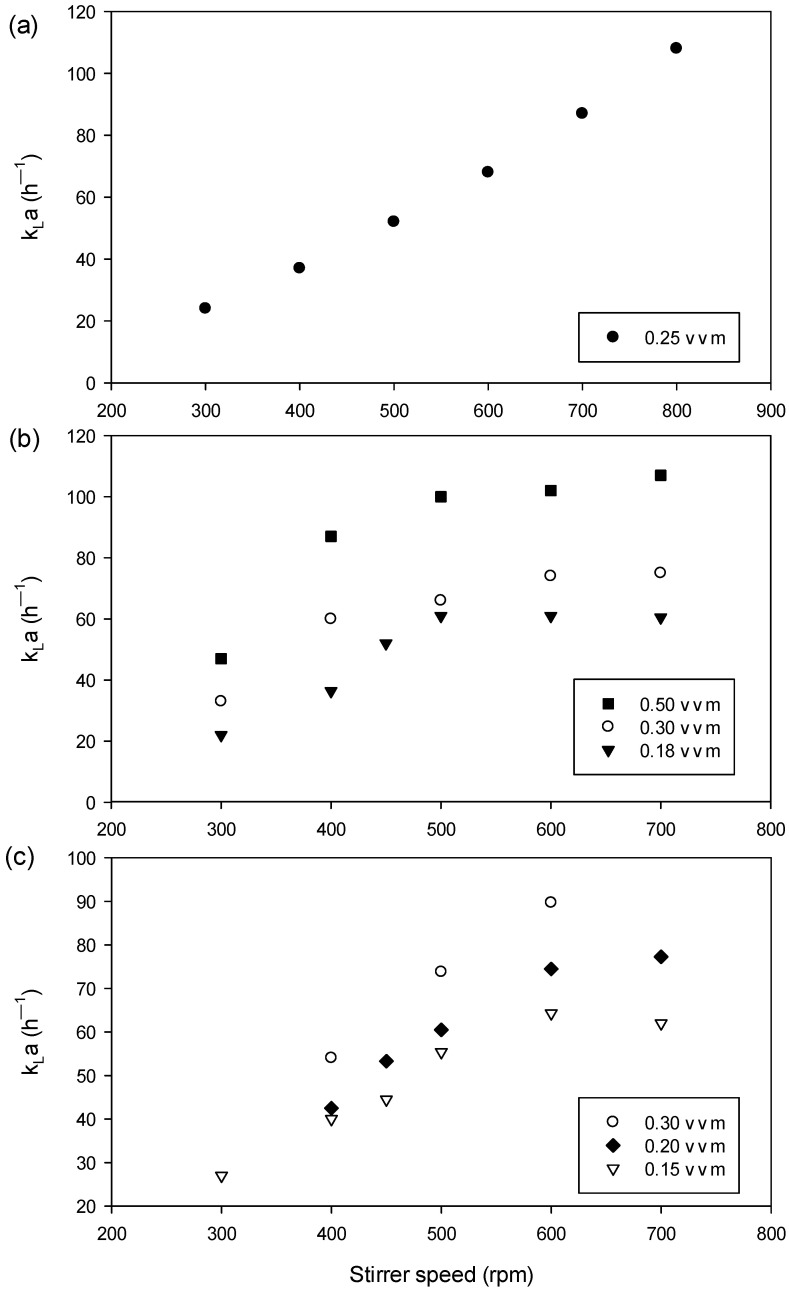
Comparison of the oxygen mass transfer coefficient (k_L_a) as a function of stirring speed (rpm) and aeration rate (vvm) in different size stirred-tank fermenter systems: (**a**) 1.5 L bench-scale, working volume (WV): 0.8 L; (**b**) 15 L bench-scale, WV: 10 L and (**c**) 75 L pilot-scale, WV: 35 L.

The 1.5 L bioreactor was carried out with 0.8 L of the culture medium. In this vessel, different values of k_L_a were evaluated at a constant aeration rate of 0.25 vvm and stirring speed values between 300–800 rpm (Figure 1). Subsequently, three different k_L_a values (lower, intermediate and high) were selected to determine the influence of pO_2_ on the cultures. The initial inoculum in the bioreactor was around a 0.1–0.2 optical density at 540 nm (OD_540nm_); samples were withdrawn in regular intervals of time to evaluate the dry cell weight, glucose, acetate and PRP measurement.

#### 2.4.4. Batch Cultures in 15 L Bench-Scale and 75 L Pilot-Scale Bioreactor Systems

Stainless steel bioreactors of 15 L and 75 L (Clean and Sterilization in Place) were conditioned and loaded using peristaltic pumps with the culture medium, 10 L and 35 L, respectively. The previously fixed k_L_a with a better biomass and PRP yield was used to establish stirring speed and aeration rate conditions. The inoculum was around a 0.1–0.2 optical density; samples were withdrawn in regular intervals timed to evaluate the dry cell weight, glucose, acetate and PRP measurement. The pH was monitored and controlled to keep at 7.0 with the addition of NaOH (5 N).

#### 2.4.5. Fed-Batch Culture in 75 L Pilot-Scale Bioreactor System

After the batch step was finalized after glucose depletion, the fresh medium was fed using a peristaltic pump (Bioengineering AG, Wald, Switzerland) using exponential flux to obtain the condition of substrate limitation. The pH of the medium was controlled at 7.0 by the automatic addition of 5 N NaOH, while pO_2_ was maintained at 30% of saturation under the aeration rate (0.18–0.20 vvm) and stirrer speed (450–900 rpm). The temperature of the culture was maintained at 30 °C. Approximately ~4.0 L of feed medium was added during the fermentation. Moreover, samples were withdrawn in regular intervals timed to evaluate the dry cell weight, glucose, acetate and PRP measurement.

### 2.5. Analytical Methods

#### 2.5.1. Biomass Measurement

A volume of 2 mL of culture broth withdrawn each hour from the culture was centrifuged at 10,000× *g* for 15 min in pre-weighed tubes. The supernatant was discarded, and the pellet was washed with 2 mL of saline solution (0.8% *w*/*w*). The supernatant was discarded, and the tubes containing the cell pellet were dried at 50 °C in the oven for 24 h, and then the mass of the dried biomass was determined after it achieved room temperature in the desiccator. The biomass was expressed in g/L, dividing the dry cell weight per sample volume.

#### 2.5.2. Glucose and Organic Acids’ Determination

The glucose and organic acids were measured by High Performance Liquid Chromatography—HPLC, Ultimate 3000 (Dionex, Sunnyvale, CA, USA). Samples were diluted five or ten times in sulfuric acid (50 mM) and filtrated in the membrane at 0.45 μm before being loaded automatically in the aminex column HPX—87H (300 mm × 7.8 mm; Bio Rad, Hercules, CA, USA) at 60 °C, with an UV (210 nm) and RI detector; the mobile phase was sulfuric acid at 5 mM, and a flow rate of 0.6 mL/min was used. The integration of chromatographic peaks to determine the concentration determination was calculated through the software Chromeleon, version 6.8 (Dionex, Sunnyvale, CA, USA).

#### 2.5.3. PRP Measurement and Molecular Weight (MW) Determination

The PRP concentration was measured by high anion exchange chromatography with pulsed amperometric detection (HPAEC-PAD). The samples were diluted in deionized water to 540 μL and 180 μL of NaOH 400 mmol·L^−1^. The mixture was incubated at 37 °C for 20 h under alkaline hydrolysis of polysaccharide and neutralized with 180 μL of 400 mmol μL acetic acid. A 100 μL glucose-6-phosphate at 100 μmol·L^−1^ was used as an internal standard. A volume of 10 μL of this mixture was injected into the anion exchange column CarboPac PA-10 coupled to pre-column AminoTrap, mounted on the ICS5000 chromatographic system (Thermo Fisher Scientific, Wilmington, MA, USA). The methodology included the gradients of NaOH and sodium acetate described by Haan et al. [30], in addition to the electrochemical potentials defined by the authors for the gold electrode. The calibration curve was plotted using a purified PRP standard at 30 mg·L^−1^ in the range of 1 to 12 mg·L^−1^.

For molecular weight (MW) determination, polysaccharide was pre-purified according to Cintra and Takagi [31], precipitating with hexadecyltrimethylammonium bromide (CTAB), and these samples were applied in a gel filtration column (2 serial TSK gel GMPW_XL_ columns) through UHPLC Ultimate 3000 (Dionex, Sunnyvale, CA, USA) at a flow rate of 0.6 mL/min, at 40 °C, and connected to the refractive index detector (RID-10A) and multi-angle light scattering (MALS)—Wyatt Technology—in series. ASTRA (Advanced System Information Tool) software from Sysinfo Lab was used to acquire and integrate data to determine MW.

### 2.6. Determination of Kinetic Parameters

#### 2.6.1. Growth Specific Rate

The growth specific rate (*μ*) was estimated from the experimental data of the biomass concentration using Equation (2) [32]:

(2) $$\mathrm{Ln}\frac{C_{DCW}}{C_{DCW-0}}=\mu_{máx}t\;$$

where *C_DCW_* is the biomass concentration (g/L) at time t, and *C_DCW_*_-0_ is the biomass concentration in the initial time (t = 0) in the fermenter.

#### 2.6.2. Conversion Factors

The conversion factors to evaluate glucose, biomass and PRP production yields were calculated according to Equations (3)–(5):

(3) YPRPGlu= CPRP− CPRP−0CGlu−0− CGlu

(4) YDCWGlu= CDCW− CDCW−0CGlu−0− CGlu

(5) YPRPDCW= CPRP− CPRP−0CDCW− CDCW−0

where *C_PRP_* is the *PRP* concentration (g/L) at time t; *C_PRP_*__0_ is the initial *PRP* concentration (t = 0); *C_DCW_* is the biomass concentration (g/L) at time t, and *C_DCW_*_-0_ is the biomass concentration in the initial (t = 0); *C_Glu_* is the glucose concentration (g/L) at time t, and *C_Glu_*_-0_ is the initial glucose concentration (t = 0).

#### 2.6.3. Productivity of Biomass and Product

For the productivities’ determination, the following Equations below were used:

(6) $$P_{DCW}=\;\frac{\left(C_{DCW}-C_{DCW-0}\right)}{t_{f}}$$

(7) $$P_{PRP}=\;\frac{\left(C_{PRP}-C_{PRP-0}\right)}{t_{f}}$$

where *C_PRP_* is the *PRP* concentration (g/L); *C_PRP_*__0_ is the initial *PRP* concentration (t = 0); *C_DCW_* is the biomass concentration (g/L); *C_DCW_*_-0_ is the biomass concentration in the initial process (t = 0), and *t_f_* is the fermentation culture time.

## 3. Results

### 3.1. Batch Cultures in 1.5 L Bench-Scale Bioreactor System and Evaluation of k_L_a Values

Figure 1 shows a determination of several k_L_a values in three sizes of bioreactor systems under different stirrer speeds and aeration rates (except in the 1.5 L bench scale). In Figure 1a, the k_L_a value increases according to stirrer speed variation in the 300–800 rpm range following a linear trend and does not reach the plateau. Figure 1b,c shows collected k_L_a values for the bioreactor of the 15 L bench and and 75 L pilot scales, respectively; in both cases, the plateau was observed beginning at 500 rpm.

In Table 2, the results of the kinetic parameters of *H. influenzae* (DCW and PRP productivity) are shown carried out at three different values of k_L_a in order to compare the biological influence of oxygen transfer. The k_L_a = 24 h^−1^ obtained low production yield; mainly, PRP productivity was affected and showed it was less than half, while, with 52 and 80 h^−1^, a non-significant difference was observed in all parameters evaluated. On the other hand, the second assay in k_L_a (52 h^−1^) under different conditions—(Intermediate)’—demonstrates that is possible to maintain a similar DCW and PRP productivity yield.

### 3.2. Scale-Up Batch Cultures in 15 L Bench-Scale and 75 L Pilot-Scale Bioreactor Systems

**Figure 2:**
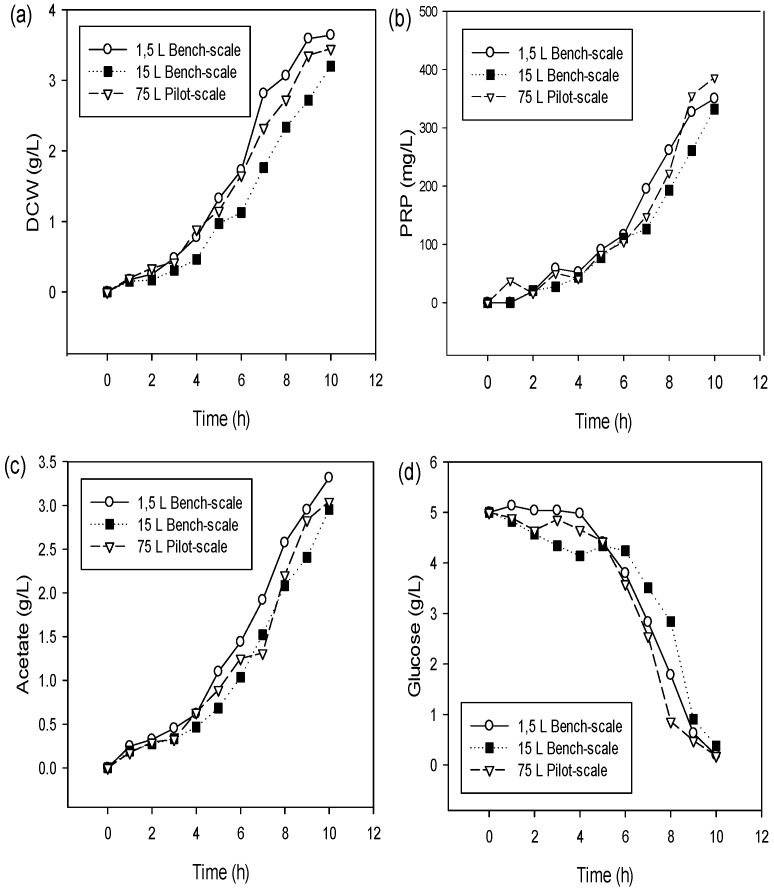
Time-course profiles of parameters’ culture of *H. influenzae* at k_L_a values equal 52 h^−1^ in three size bioreactor systems: 1.5 L^−1^ bench–scale, WV: 0.8 L^−1^, OC: 500 rpm and 0.25 vvm; 15 L bench-scale, WV: 10 L^−1^, OC: 450 rpm and 0.18 vvm; and 75-L^−1^ pilot-scale, WV: 35-L, OC: 500 rpm and 0.15 vvm. (**a**) Dry cell weight (DCW); (**b**) PRP production; (**c**) Sodium acetate; (**d**) Glucose consumption. WV: Working Volume, OC: Operative Conditions.

Figure 2 showed a comparative profile of growth, product and sub-product formation and substrate consumption of *H. influenzae* in three sizes of bioreactor systems working at k_L_a = 52 h^−1^. In the case of the 15 L bench-scale and 75 L pilot-scale bioreactor systems, the operative conditions were established at 0.18 vvm/450 rpm and 0.15 vvm/500 rpm, respectively. Profiles presented similar behavior in the three fermentations, DCW (Figure 2a), PRP production (Figure 2b), acetate formation (Figure 2c) and glucose consumption (Figure 2d). In the case of DCW production (Figure 2a), the exponential growth phase was a linear behavior in 4–8 h and reached the stationary growth phase at 9 h; at the same time, a decrease in glucose concentration occurred, while the PRP production curve and acetate formation (2–3 g/L) showed an association at the cell growth.

Table 3 presents a comparative productivity yield (*PRP* and *DCW*) and conversion factors (*Y_DCW/Glu_*, *Y_PRP/Glu_* and *Y_PRP/DCW_*) of *H. influenzae* in three sizes of bioreactor systems working at k_L_a = 52 h^−1^ during 9 h. The differences obtained in the three cultures were lower than 14% in terms of productivity and conversion factors between scales, corroborating the observation in Figure 2. Although DCW and PRP productivity in the 15 L bench-scale was 4% lower than other sizes, it is possible to assure an efficient establishment of k_L_a as the criterion scale–up.

### 3.3. Fed-Batch Culture in 75 L Pilot-Scale Bioreactor System

**Figure 3:**
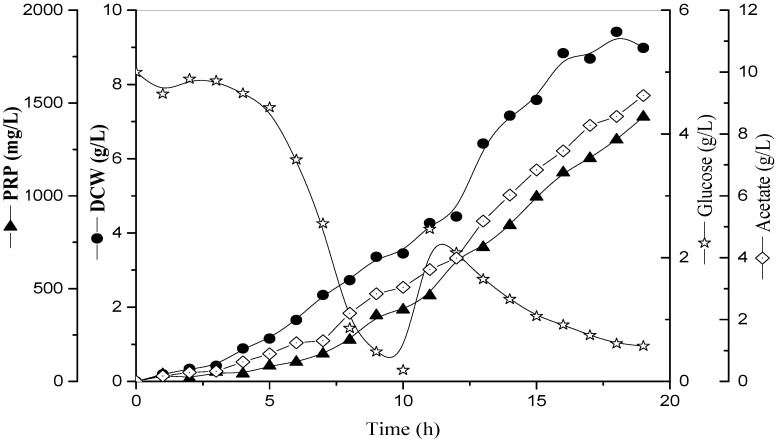
Time-course profiles of cell growth (*DCW*), glucose consumption, *PRP* production and acetate formation by *H. influenzae* at k_L_a values equal 52 h^−1^ under two operation modes in 75 L pilot-scale, WV: 35 L^−1^. Batch phase: 500 rpm and 0.15 vvm; Fed-batch phase: pO_2_ at 30% of saturation. WV: Working Volume. Fed-batch phase was started at 10 h using external peristaltic pump.

Figure 3 shows the culture of *H. influenzae* in two stages: batch (under k_L_a 52 h^−1^ constant) and fed-batch (under 30% pO_2_ constant). In order to avoid the oxygen transfer limitation during the second stage due to the increase in biomass, the oxygen supply was modified at 10 h while the feeding substrate was started. The *H. influenzae* maintained stable growth characteristics under this strategy; a delay in growth was observed since 17 h of the culture. Moreover, *PRP* production and acetate formation showed an association at the cell growth. At the final process, the total productions of *PRP* and *DCW* reached were 1.43 g/L and 9.0 g/L, respectively.

### 3.4. Molecular Weight (MW) of Produced PRP

Table 4 shows the molecular weight (*MW*) of *PRP* produced at the end of the culture. All fermentations were carried out during 10 h while the fed-batch in the pilot-scale was maintained for 9 h The *PRP* produced at k_L_a 52 h^−1^ presented a similar *MW* (in average 347.2 kDa) independent of the size of the bioreactor system, indicating that the length of *PRP* was maintained when the oxygen supply strategy was constant. Moreover, the MW of *PRP* obtained at the final stage of the fed-batch in pilot-scale process (375 kDa) indicated that sufficient oxygen supply will positively influence the length of *PRP*.

## 4. Discussion

### 4.1. Batch Cultures in 1.5 L bench-Scale Bioreactor and Evaluation of k_L_a Values

The successful scale-up of aerobic processes depends fundamentally on the oxygen mass transfer to the cell present in the fermentation broth, which depends on the aeration rate and stirring speed [27,33]. Thus, the stirred tank bioreactor is commonly used because it provides efficient mixing with high values of heat, nutrients and oxygen transfer rates [25,33]. The linear profile of k_L_a obtained using the 1.5 L bioreactor was similar to the one obtained by Averkina et al. [34] under a stirrer range of 50–300 rpm. Therefore, there is a direct influence of agitation on gas; it is dispersed, caused by the impeller with a varying efficiency for breaking bubbles and produces the increase in the gas liquid interface area and residence time into the medium culture [26,33,35], while the k_L_a profile obtained in 15 L and 80 L was similar to that reported by Shin et al. [23] under evaluation of a different stirrer and aeration rate.

On the other hand, fermentation conditions established at the small scale allow for reducing costs associated with experimentation [16]. For this reason, the 1.5 L bioreactor was used to study the effects produced by k_L_a; it was investigated experimentally using three different values (low: 24 h^−1^, intermediate: 52 h^−1^ and high: 80 h^−1^) obtained in 300, 500 and 700 rpm of stirring speeds, respectively. The generated turbulence at the lower speed rate (300 rpm) was not enough to trap and hold up the air bubbles, and consequently the performance of the oxygen mass transfer may not be noticeably efficient [36]. Lower stirrer speeds (around 300 rpm) result in reduced mixing in the medium and poor oxygen supply to the microorganism [33]. In the value of k_L_a (24 h^−1^), our results showed lower *DCW* (0.22 g/L.h) and *PRP* (0.01 g/L.h) productivity because the environmental condition could affect gene expression and/or regulation of enzymes involved in the polysaccharide synthesis [19] and reduced the uptake of the substrate to growing. Conversely, between the 500 and 800 rpm range would be beneficial to promote the liquid turbulence necessary to generate the high-rate dissolved oxygen [35]. The fermentations carried out with intermediate and high k_L_a values (52 h^−1^ and 80 h^−1^) showed not great differences; in *DCW* (0.36 and 0.40 g/L.h) and *PRP* (0.04 g/L.h in both cases) productivity values, respectively, were obtained because the availability of oxygen was enough for the microorganism in both cases. Therefore, these conditions allowed the microbial growth for utilizations in biomass building. The catalytic activities were fully utilized from that oxygen level and could be maintained in the immediate vicinity of the cells [37]. According to Rodrigues et al. [38], there is a positive influence of the high kLa value (above 84 h^−1^) in biomolecule production. In order that k_L_a and the stirring speed are factors that influence physical and biological characteristics (i.e., growth of microorganism, product concentration) [39] and oxygen transfer, they may cause strong effects in product formation by influencing metabolic pathways and changing metabolic fluxes [25,26]. Based on the results obtained, the k_L_a = 52 h^−1^ value was selected for the further experiments; the efficiency of this k_L_a value was corroborated by an additional assay obtained at 700 rpm and 0.15 vvm (Table 2). Mainly, operation under 500 rpm presented several advantages such as lower energy expenditure, reduced heat generation, avoiding shear damages imposed on the cells at vigorous agitations, preventing morphological and physiological changes, avoiding growth rate modifications and product inactivation [23,37,40,41].

### 4.2. Scale-Up Batch Cultures in 15 L Bench-Scale and 75 L Pilot-Scale Bioreactor Systems

The principal objective of the scale-up carried out in the bioreactor is to maintain or to improve yield in both large and small tanks [16,21]. However, the heterogeneities in large-scale bioreactors may amplify the impact of physical interferences, negligible in the bench scale [15]. For example, changes in operational conditions between different sizes of bioreactors are caused due to small differences in geometry, configuration and size correlations of components affecting the biological behavior of bacteria, but these smaller changes are not enough to influence the culture process significantly. In this sense, the knowledge of cell behavior is necessary to predict the influence of environmental factors and product yield in scale-ups [27,30]. Scale-up criteria provide a comparable microbial behavior between small- and large-scale bioreactors [15]. According Kadri et al. [42], the oxygen transfer capacity applied in the bioreactor scale-up process allowed for obtaining better bacterial growth and higher biomolecule production. Therefore, it is necessary to consider variations due to effects of oxygen depletion by absorption, hydrostatic head, gas phase back mixing and heterogeneities in the liquid phase (stagnant zones) [43]. Based on our previous results, an optimal value of k_L_a (52 h^−1^) was defined in the 1.5 L bioreactor; we used these results to reach an effective scale-up of the *PRP* production process with the purpose of successful production in the 15 L bench-scale and 75 L pilot-scale bioreactor systems. The profiles in Figure 2 showed that the kinetic of growth, *PRP* production, glucose consumption and acetate formation were similar in three sizes of bioreactor systems. According to Gameil et al. [16], this fact indicated that the constant k_L_a remained the best option, giving high yield upon the scale-up. In this sense, due to the microbial behavior, both the bench-scale and pilot-scale are comparable, and the scale-up is considered a success.

Fermentation carried out in the 75 L pilot-scale bioreactor showed a higher yield profile in the production of *PRP* and *DCW* in comparison with 1.5 L and 15 L bioreactors (Table 3), although, commonly during scaling-up, a 10–30% of reduction in performance associated with heat and mass transfer issues was observed [14]. On the other hand, differences in *PRP* production in fermentations could be explain by a spontaneous loss of capsular expression; this phenomenon occurs in several *H. influenzae* type b strains with a rate of 0.1–0.3% per generation [44]. Moreover, reduced *PRP* production was caused by insufficient oxygen available in the medium because the regulation and expression of genes involving *PRP* biosynthesis are affected by the pO_2_ concentration [19], and under our operational conditions were observed pO_2_ values less than 5% during various hours in the exponential growth phase (data not shown).

### 4.3. Fed-Batch Culture in 75 L Pilot-Scale Bioreactor System

In the pilot scale used in this study, it was also carried out using a fed-batch stage after total depletion of glucose at 10 h of the culture; also, the rapidly increasing O_2_ value presented an observable peak in the probe profile and could be used such as criteria for detection of substrate exhaustion in the logarithmic growth phase [45,46]. Our previous tests showed an important reduction in oxygen levels during the batch operation mode (data not shown). This observation was explained due to limiting solubility of oxygen in water (and fermentation media), medium composition changes and increases in the microorganism concentration [16,25]. However, in many microbial processes, the values of dissolved pO_2_ played a decisive role in bacterial metabolism [47]; an oxygen deficiency will lead to either cessation of aerobic growth or a reduction in substrate uptake efficiency and production of undesirable by-products [16,48]. Subsequently, a constant pO_2_ level can be maintained during the fed-batch mode operation to reach a balance between oxygen consumption and supply. In fact, fed-batch strategies include a constant dissolved oxygen value [45]. For instance, Shin et al. [23] recommended a pO_2_ value around 20% for some microbial cells in culture, while several previous studies in *H. influenzae* established pO_2_ at 30% of saturation [12,19,31], although, Takagi et al. [19] suggested that this microorganism could be grown at lower levels of dissolved oxygen, because no significant difference was found in the *PRP* production using pO_2_ at 10 and 30% of saturation. In order to avoid these negative effects, we decided to use pO_2_ at 30% of saturation during the fed-batch stage.

The growth was increased since 10 h accompanied *PRP* production and acetate formation, indicating that not oxygen limitation was observed in the culture. It was confirmed that *PRP* synthesis is totally associated with the cell growth, according to the model described by Luedeking-Piret and reported by Takagi et al. [19] for *H. influenzae* type b. The controlling glucose feed rate was started in order to avoid the accumulation of metabolites such as acetate formation [12,49]. Acetate in the protonated form (acetic acid) can diffuse freely across cell membranes, resulting an accumulation of acetate anions and protons in the cell cytoplasm and a reduction in intracellular pH of 4.76. Moreover, to maintain the membrane potential, protons in excess have to be expelled, causing an energy expenditure detrimental to growth [50,51]. Thus, at an acetate concentration above 8 g/L, a pH of 7.4 showed inhibitory effects in *E. coli* (reduction of growth rate from 0.75 to 0.4 h^−1^) [51]; according to our results, the acetate concentration reached 8.2 g/L from 17 h, correlating with the plateau in the growth curve.

In order to get the desired product, *PRP* production values obtained were 7 and 17% more than those reported by Merrit et al. [12] and Arsang et al. [4], respectively (Table 5); it is possible to explain this result because high biomass production was reached in our process. These results are just comparable with Da Silva et al. [52] whose productions were 1.69 g/L *PRP* and 15 g/L *DCW*, though this process was carried out in the bench-scale bioreactor. Therefore, the fed-batch can be affected positively in the productivity of the desired products and must be conducted in order to improve the cultivation process produced in the effective scale-up of the *PRP* fermentation process. Moreover, this is the first available study of a scale-up applying the k_L_a criterion for the *H. influenzae* culture.

### 4.4. Molecular Weight (MW) of Produced PRP

*PRP* is a linear copolymer whose size or molecular weight (*MW*) is considered to be an important factor in the efficacy of Hib vaccines [3,5]. A high *MW* is beneficial to the development process of conjugated vaccines in accordance with the WHO requirement [53,54]. Due to controlled fragmentation, depolymerization with sodium metaperiodate allowed for obtaining defined and control-sized polysaccharides that could be used for the vaccines’ production [3,55]. On the other hand, the native *PRP* size is determined by several factors such as bacterial strains, medium composition (carbohydrate source and carbon/nitrogen ratio; content of amino acids, vitamins, phosphate, mineral, etc.) and culture conditions (pH, temperature, oxygen tension, etc.) [56] in order to consider that all fermentation performances were maintained under the k_L_a criterion scale-up process. The results in Table 3 showed that the *PRP* obtained presents an average *MW* about 347.2 kDa, while the *MW* of native *PRP* reported in a previous study was found between 450 and 600 kDa [55]. In spite of the *PRP* produced in our study, it can still be successfully used for vaccine production because studies about the immunological response analysis indicate that the size-reduced oligosaccharide of about 10 kDa or with an average of 20 repeating units may be better able to elicit T-cell dependent antibody responses [5,55].

## 5. Conclusions

A simple protocol for scaling-up based on a constant k_L_a in the batch culture was developed until a 75 L pilot-scale was achieved. The overall scale-up and process transfer performance was acceptable where similar bioreactor yields, productivity and kinetic behaviors were maintained across three sizes of fermenters systems. This process, based on the batch stage operated under two stages in different oxygen modes, was successful in producing *PRP* by *H. influenzae* in the pilot-scale; this fact is very important because the operational conditions and hydrodynamic/mixing time are very similar to those used at the industrial scale. Therefore, these results confirm that the performance of this strategy can be scaled up and could be beneficial for future bioprocess operations that may lead to a higher productivity and less operative cost.

## Figures and Tables

**Table 1:** Comparison of bioreactor systems’ specifications.

Specifications	Bioreactors—Nominal Size		
	1.5-L	15-L	75-L
Brand	Infors-HT	Bioengineering	Bioengineering
Impeller	Rushton 2 impellers with 6 blades each. Diameters: 38 mm	Rushton 2 impellers with 6 blades each Diameter: 80 mm	Rushton 2 impellers with 6 blades each Diameter: 163.5 mm
Drive	Magnetic	Bottom drive with belt shaft	Bottom drive with belt shaft
Tank diameter—ID (mm)	90	200	400
Type of sparge	Ring sparger	Ring sparger	Ring sparger
Working volume	0.8-L	10-L	35-L

**Table 2:** Comparison of kinetic parameters obtained from cultures in 1.5 L bioreactor performed with *H. influenzae* using different k_L_a values.

k_L_a (h^−1^)	Stirring Speed (rpm)	Aeration Rate (vvm)	Time (h)	Productivity		μ (h^−1^)
				DCW (g/L.h)	PRP (g/L.h)	
24 (Low)	300	0.25	12	0.22	0.01	0.31
52 (Intermediate)	500	0.25	10	0.38	0.04	0.44
52 (Intermediate)’	700	0.15	10	0.36	0.04	0.43
80 (High)	700	0.25	9	0.40	0.04	0.46

**Table 3:** Comparison of cultures’ yields and conversion factors obtained during the scale-up process of *H. influenzae* using k_L_a criterion performed in 1.5 L bench-scale, 15 L bench-scale and 75 L pilot-scale bioreactor systems during 10 h of culture.

Total Volume (L)	Working Volume (L)	DCW (g/L)	PRP (g/L)	DCW (g/L.h)	PRP (g/L.h)	*Y_DCW/Glu_* (g/g)	*Y_PRP/Glu_* (g/g)	*Y_PRP/DCW_* (g/g)	μ (h^−1^)
1.5	0.8	3.8	0.36	0.38	0.04	0.8	0.08	0.10	0.44
15	10	3.2	0.33	0.32	0.03	0.7	0.07	0.10	0.46
75	35	3.4	0.38	0.34	0.04	0.7	0.08	0.11	0.49

**Table 4:** Comparison of molecular weight—MW (kDa) of capsular *PRP* from *H. influenzae* obtained during scale-up process using k_L_a criterion (52 h^−1^) carried out in three size bioreactor systems.

Bioreator Size (L)	Operational Mode	Conditions	Time (h)	MW (kDa)
1.5	Batch	0.25 vvm- 500 rpm	10	355.5
15	Batch	0.15 vvm -450 rpm	10	337.4
75	* Batch	0.18 vvm -450 rpm	10	348.6
75	* Fed-batch	pO_2_ 30%	9	375.0

* These cultures were part of the same process.

**Table 5:** Previous studies in pilot and large scale-up processes, conditions and production (*DCW* and *PRP*) by *H. influenzae* using bioreactor systems.

Volume	Medium Composition (g/L)	Culture Conditions		DCW (g/L)	PRP (g/L)	Ref.
		Batch	Fed-Batch			
FV: 50 L WV: ND FM: ND	glucose 6.0; yeast extract 2.5; casamino acids 10; NaH_2_PO_4_ 0.1 M; hemin 0.03; NAD 0.015.	Inoculum 0.5%, pH 7.3, 36.5 °C, 30% (pO_2_) [0.6–0.8 vvm, 400–900 rpm], 22 h		5.2	1.16	[4]
FV: 500 L WV: 370 L FM: 40 L	glucose 10.0; yeast extract; casamino acids 10; Na_2_HPO_4_ 12.4; NaH_2_PO_4_.H_2_O 1.8; hemine chloride 0.04; NAD 0.02	pH 7.3, 36.5 °C, 50% pO_2_ [0.6–0.8 vvm, 400–900 rpm], 14.5 h		6.0	1.3	[12]
FV: 75 L WV: 35 L FM: 4 L	described on item 2.2	pH 7.0, 37 °C, k_L_a 52 h^−1^, [0.18 vvm, 450 rpm], 10 h	30% (pO_2_) [0.18–0.20 vvm, 400–900 rpm], 9 h	9.0	1.4	This article

FV: Fermenter Volume; WV: Working volume; FM: Feed Medium volume; ND: non-determinate.

## Data Availability

Not applicable.
